# Emotion recognition in autism spectrum condition during the COVID-19 pandemic

**DOI:** 10.1177/13623613231203306

**Published:** 2023-10-26

**Authors:** Tim Schnitzler, Christoph Korn, Sabine C. Herpertz, Thomas Fuchs

**Affiliations:** Heidelberg University, Germany

**Keywords:** autism spectrum disorder, COVID-19, emotion recognition, eye tracking

## Abstract

With the widespread use of masks in the COVID-19 pandemic, it is crucial to understand how emotion recognition is affected by partial face covering. Since individuals with autism spectrum condition often tend to look at the lower half of the face, they are likely to be particularly restricted in emotion recognition by people wearing masks, since they are now forced to look at the upper half of the face. This study compared the recognition of basic and complex emotions in individuals with and without autism spectrum condition, when faces were presented uncovered, with face masks, or with sunglasses. We also used eye tracking to examine group differences in gaze patterns during emotion recognition. Individuals with autism spectrum condition were less accurate at recognizing emotions in all three conditions. Averaged across the three stimulus types, individuals with autism spectrum condition had greater difficulty recognizing anger, fear, pride, and embarrassment than control group. There was no group difference in emotion recognition between the three conditions. However, compared to individuals without autism spectrum condition, there was no evidence of either gaze avoidance or preference for the mouth region. Our results suggest that emotion recognition is reduced in individuals with autism spectrum condition, but this is not due to differences in gaze patterns.

**Lay Abstract:**

In the COVID-19 pandemic, wearing face masks became mandatory to prevent the spread of the virus. However, they restrict the ability to recognize emotions to the upper part of the face. Since individuals with autism spectrum condition often tend to look at the lower half of the face, they may be particularly restricted in emotion recognition by people wearing masks, since they are now forced to look at the upper half of the face. The current study compared the recognition of facially expressed emotions between individuals with and without autism spectrum condition. Each photo was shown in three types, once uncovered, once with face mask, and once with sunglasses. Our results revealed a reduction in accuracy of individuals with autism spectrum condition at recognizing emotions in all three stimulus types and exhibited more difficulties distinguishing anger, fear, pride, and embarrassment. During the emotion recognition task, there was no difference in which facial areas the groups looked at. We did not find evidence that the disadvantages of individuals with autism spectrum condition in emotion recognition were due to looking at different areas of the face.

## Introduction

The worldwide outbreak of COVID-19 and the associated restrictions of social life have dramatically changed the way people interact. In particular, face masks became prevalent and even mandatory to prevent the spread of the virus (MacIntyre & Chughtai, 2020). These face coverings have a huge impact on the visibility of facial expressions (Carbon, 2020; Fitousi et al., 2021), as information-rich areas like the mouth (Blais et al., 2012) are covered and only the upper part of the face can be used to discern another person’s emotional expression. But emotion recognition is crucial for interactions and social–emotional reciprocity (Scherer & Ekman, 1984; Tanaka, 2001), as the facial expression of emotions is one of the most important ways of communicating with other persons (Mehrabian, 1968). During catastrophes or pandemics, vulnerable populations, such as individuals with autism spectrum condition (ASC) are particularly affected, as they need to change their daily routines and to adapt their compensation mechanisms in social interactions, which is associated with anxiety (Baweja et al., 2022; Mutluer et al., 2020).

Usually, gaze in social interaction is mostly directed at the eyes of another person (Schyns et al., 2002). But individuals with ASC are often considered to favor information from the mouth region and avoid the eyes (Spezio et al., 2007), which is explained in two ways: first, individuals with ASC actively avoid eye contact, because they experience it as aversive (Tanaka & Sung, 2016), and second, because they experience facial expressions as less salient (Senju & Johnson, 2009). Assuming that individuals with ASC spend less time looking at the eyes, it is relevant to examine the impact of mask covered faces on facial emotion recognition (FER) in individuals with ASC (Amaral & de Vries, 2020).

The eye and mouth areas are important features for recognizing basic individual emotions. Sadness, anger, and fear are mainly recognized from the eyes (“upper-face” expressions), while happiness and disgust are recognized mainly from the mouth area (“lower-face” expressions; Bombari et al., 2013; Wegrzyn et al., 2017). Complex emotions like pride and embarrassment differ from the basic ones in that the expression diverges depending on a culture-specific interplay between eye and mouth regions (Russell & Paris, 1994). Recent studies on the impact of face masks on emotion recognition in non-autistic individuals have shown a decrease in accuracy of FER due to masks (Carbon, 2020; Kastendieck et al., 2022). With exception of faces with a fearful and neutral expression, the recognition of facial expressions showing disgust, happiness, anger and sadness was impeded by wearing a mask (Carbon, 2020). As fear is primarily detected by the eyes (Bombari et al., 2013; Wegrzyn et al., 2017), the facial features from the upper half of the face could be sufficient to correctly detect it despite wearing a mask. If the eyes are covered, for example, with sunglasses, FER is less impaired than with a face mask (Noyes et al., 2021; Roberson et al., 2012).

The studies that have already dealt with FER in ASC, mostly based on photos, revealed inconsistent results. Some studies claim that, in contrast to non-autistic persons, the recognition of most basic emotions (Philip et al., 2010; Uljarevic & Hamilton, 2013) or only specific emotions like fear (Humphreys et al., 2007; Pelphrey et al., 2002), sadness (Boraston et al., 2007), or anger (Keating et al., 2022) is reduced. Yet, other studies revealed no group difference (Castelli, 2005; Jemel et al., 2006). Conflicting results were also found for complex emotions (Black et al., 2020; Golan et al., 2006). For these discrepancies various factors were mentioned: individuals with ASC may not use their cognitive compensation strategies to perceive emotions correctly (Santos et al., 2008) when there is a time limit (Clark et al., 2008; Nagy et al., 2021) or depending on the experimental task (Harms et al., 2010). Recently, it was claimed that it is not autism per se, which is linked to reduced FER in ASC but rather alexithymia (Bird & Cook, 2013; Ola & Gullon-Scott, 2020). Alexithymia is characterized by difficulties in identifying emotions (Nemiah et al., 1976), which is often comorbid with ASD (Kinnaird et al., 2019). This hypothesis was recently investigated by a study, which compared the impact of masks on FER in individuals with and without ASC (Gehdu et al., 2023). High-alexithymic autistic participants had greater difficulty recognizing masked and uncovered stimuli than low-alexithymic autistic participants and non-autistic participants, whereas, there was no difference between low-alexithymic autistic and non-autistic participants.

As atypical scanning patterns on emotional faces and diminished eye contact are common features in individuals with ASC (Kliemann et al., 2010; Spezio et al., 2007), their ability to categorize emotions through masked faces may be especially reduced. This led us to hypothesize that covering the lower half of the face may have a greater impact on individuals with ASC in comparison to non-autistic persons. In this study, we investigate the effect of covering facial regions with a mask or sunglasses on FER in individuals with and without ASC. In addition, we examine gaze patterns during the emotion recognition task using eye tracking. This allows us to explore to what extent group differences in FER are due to different scanning paths.

## Materials and methods

### Participants

The recruitment of participants took place between August and December 2021 in Heidelberg, Germany. During this period, masks (mostly FFP2 masks) were required to be worn outdoors, if a safety distance of 1.5 m could not be maintained, and always indoors by everyone over the age of six (regardless of vaccination status). Within this period, we tried to include as many individuals with ASC as possible. A fixed time period was therefore set, as mask wearing regulations frequently changed, and it was unclear how long the obligatory mask protocol would last. Non-autistic participants were matched with respect to age, gender, and verbal IQ.

As a post hoc power analysis is controversial (Dziak et al., 2020), it is also not strictly valid to conduct an *a priori* power analysis after the results, but it never less gives an indication of how many participants are needed for a specific effect size. We conducted such an analysis in retrospect using G*Power version 3.1.9.7 (Faul et al., 2007) for the interaction group × condition. We calculated the sample size assuming a small effect size (0.2). Results indicated the required sample size to achieve 85% power at a significance criterion of α = 0.05, was a total sample size of *N* = 48 for a repeated-measure analysis of variance (ANOVA) with within and between interaction. We included 36 participants each with and without ASCs with no known history of neurological or psychiatric disease or pervasive developmental disorder. Thus, the obtained sample size per group seems adequate to test the group by condition hypothesis.

Six additional non-autistic subjects participated in a pilot study to determine the stimulus duration for the FER task. All ASC participants met *Diagnostic and Statistical Manual of Mental Disorders, 5th Edition* (*DSM*-V) diagnostic criteria for ASC (American Psychiatric Association, 2013). The diagnosis was made in advance of the study through psychiatrists who are specialized in ASC. Participants with ASC were recruited from our hospital as well as from surrounding autism centers. Non-autistic participants were recruited by posting announcements (Table 1).

**Table 1.** Demographic variables and diagnostic scores.

	ASC	Non-autistic	*p*
Age	28.08 ± 9.09	28.44 ± 7.61	0.856
Gender (female/male)	7/29	7/29	1.0
MWT	29.94 ± 2.7	30.11 ± 3.08	0.808
AQ	34.83 ± 7.17	12.31 ± 6.09	< 0.001
TAS-26	50.56 ± 8.65	35.92 ± 7.89	< 0.001

Values are given as mean ± standard deviation. *p*-values reflect levels of significance from independent samples *t*-tests. ASC: autism spectrum condition; MWT: multiple choice vocabulary test; AQ: autism spectrum quotient; TAS-26: Toronto Alexithymia Scale—26.

All participants had normal or corrected to normal vision at testing time, were native German speakers and gave written informed consent. They received no payment for participating in the study.

### Stimuli

**Figure 1.**
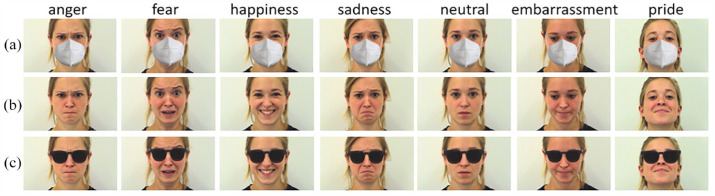
A model showing seven different emotions wearing a mask (a), uncovered (b) and wearing sunglasses (c). Original stimuli from Amsterdam Dynamic Facial Expression Set (Hawk et al., 2008).

Stimuli were selected from the Amsterdam Dynamic Facial Expression Set, which has been validated for emotion recognition rates (Hawk et al., 2008). Written permission to modify the photos using *Adobe Photoshop*^®^ was given by the authors who developed this database. We used photographs depicting the basic emotions of anger, happiness, sadness, and fear, as well as neutral state and the complex emotions of pride and embarrassment. All emotions were posed by five female and seven male models aged between 18 and 25. All models utilized the same facial action units to portray an emotion (Ekman & Friesen, 1978; Keltner, 1995; Tracy & Robins, 2004). The emotional images were selected from the end point of the dynamic set (point of most intense muscular contraction; Hawk et al., 2008). These emotions were chosen to contrast basic and complex emotions, as well as “upper-face” and “lower-face” expressions. Each emotion picture was presented in three different conditions: uncovered, partially covered with a face mask, or sunglasses (Figure 1). A total of 252 photos were presented (3 conditions × 7 emotions × 12 models). All photos were presented in color using Presentation^®^, neurobehavioral systems. The size of photographs (distance between eyes and mouth: 7.5 cm) was based on Gamer and Büchel (2009). The distance between the eyes and mouth was 5.7 degrees of visual angle. In the vertical direction, the visual angle of the photo was 21.25° and in the horizontal one 35°.

### Emotion recognition task

**Figure 2.**
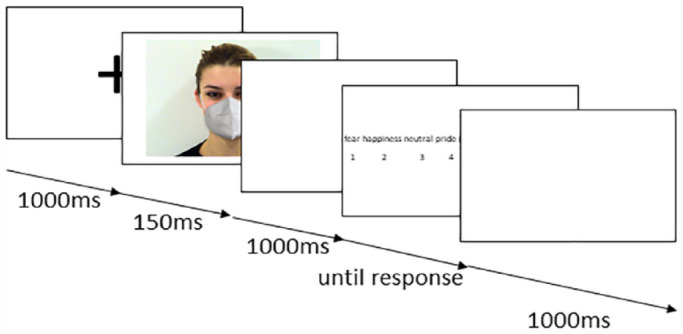
Illustration of the trial structure that was used in the experiment.

The experiment was based on a 7 × 3 × 2 design with EMOTION (anger, happiness, sadness, fear, neutral, pride, and embarrassment) and CONDITION (uncovered, mask, and sunglasses) as within-subject factors and with GROUP (ASC and non-autistic) as the between-subjects variable. Each trial started with a fixation cross (1 s), placed between eyes and mouth of the subsequent photo (see Figure 2). Afterwards, one photo was presented for 150 ms (duration was chosen to capture immediate perception rather than cognitive compensation strategies). Previous studies have shown that despite brevity, emotions can be detected (Clark et al., 2008; Kliemann et al., 2010). The stimuli were pseudo-randomized, so that, the same condition was never presented twice in a row. After a blank white screen (1 s), participants had to decide which emotion they had seen, using the buttons 1–7 on a standard keyboard (report rate: 1000 Hz; Logitech^®^). All seven emotions were listed in the middle part of the screen according to the alphabet. Under each emotion, a number was written and the respective button with the correct number had to be pressed. Since there was no time limit, the emotion labels were presented until the participant made the decision. No feedback was provided. After the categorization, there was a break (1 s).

### Questionnaires

Participants had to fill in several questionnaires. Autistic traits were measured via the autism spectrum quotient (AQ; Baron-Cohen, Wheelwright, Skinner, et al., 2001). Levels of alexithymia were assessed by the Toronto Alexithymia Scale—26 (Taylor et al., 1985, 1990). Verbal IQ levels were tested by a multiple choice vocabulary test (Lehrl, 2005). We developed a questionnaire (*emotion recognition questionnaire*; see Supplemental Material) to assess the subjective change in emotion recognition and scanning paths on face and body during COVID-19 pandemic.

### Eye tracking

The eye tracker (60 HZ, *Tobii Pro X2*) was placed under a 21.5-inch screen that did not require fixation to participants’ heads. Participants were seated 70 cm from the eye tracker toward a screen on a height-adjustable chair. The illumination of the testing room was kept constant. Gaze direction and pupil diameter for both eyes were recorded throughout the entire experiment using the manufacturer’s software at a sampling rate of 60 Hz. We used the 5-point calibration implemented in the manufacturer’s software for calibrating gaze direction once at the beginning of the experiment. Pupil data were analyzed using MATLAB. We excluded participants with less than 200 trials for which pupil data were available. We therefore excluded four individuals with ASC and five individuals without ASC. Overall, we could analyze a mean of 249.9 trials per participant (*SD* = 5.6; minimum = 223).

Gaze data were *z*-normalized. To test for differences between the three CONDITONS and the two GROUPS, we compared the average vertical gaze positions between three time windows of 500 ms duration. The first time window served as a control condition and started before the faces were shown; the second time window started with stimulus onset; and the third time window lasted from 500 to 1000 ms after stimulus onset.

### Community involvement

The idea for the study arose from conversations with autistic individuals and subsequently they were involved in designing the paradigm. In conjunction with collaborating autism centers, results were shared within the community.

## Results

### Emotion recognition in ASC individuals with partly covered face stimuli

The accuracy in both groups was much higher in the uncovered condition than in the other ones (see Figure 6). In the uncovered condition, emotional accuracy in both groups was at least 65%, except pride that was 35.8% accurate in the ASC group. Comparing the two groups, non-autistic participants had descriptively higher recognition accuracies for all emotions of the three conditions except happiness for the stimuli with sunglasses.

A 2 × 3 × 7 repeated-measures mixed-factorial ANOVA was conducted with GROUP as a between-group variable, and CONDITION and EMOTION as a within-group variable. We found significant main effects of CONDITION (*F*_(2, 69)_ = 102.31841, *p* < 0.001, η_*p*_^2^ = 0.748) and EMOTION (*F*_(6, 65)_ = 37.555, *p* < 0.001, η_*p*_^2^ = 0.776) as well as significant double interactions CONDITION × EMOTION (*F*_(12, 59)_ = 44.270, *p* < 0.001, η_*p*_^2^ = 0.900) and EMOTION × GROUP (*F*_(6, 65)_ = 3.097, *p* = 0.01, η_*p*_^2^ = 0.222). The predicted interaction effect between CONDITION and GROUP failed to approach statistical significance (*F*_(2, 69)_ = 0.611, *p* = 0.546, η_*p*_^2^ = 0.017). The triple interaction CONDITION × EMOTION × GROUP reached significance (*F*_(12, 59)_ = 3.841, *p* < 0.001, η_*p*_^2^ = 0.439).

We calculated three 7 × 2 ANOVA’s before conducting *t*-tests for the significant triple interaction, each for the three conditions, with GROUP as a between-group variable and EMOTION as a within-group variable.

For the masked stimuli, we found a significant main effect EMOTION (*F*_(6, 65)_ = 35.880, *p* < 0.001, η_*p*_^2^ = 0.768), the interaction EMOTION × GROUP failed to reach statistical significance (*F*_(6, 65)_ = 35.880, *p* = 0.250). There was a significant GROUP effect (*F*_(1, 70)_ = 19.728, *p* < 0.001, η_*p*_^2^ = 0.111), revealing a reduced emotion recognition in individuals with ASC compared to non-autistic participants (mean ± SEM for ASC: 59.1 ± 7.7; non-autistic: 72.4 ± 1.5). A further analysis of the significant main effect EMOTION (collapsed across group) revealed that fear was recognized significantly better by all participants than happiness, sadness, pride, and embarrassment (see Table 2). Happiness was detected better than sadness and pride, while neutral was detected better than pride and sadness. Pride was recognized worse than embarrassment and anger, but anger better than sadness.

**Table 2.** Results of the two-tailed, one-sample *t*-tests (Bonferroni-corrected) performed on the main effect EMOTION (collapsed across group) for face-masked stimuli.

	*T*	*df*	*p*
fear–happiness	3.874	71	0.005**
fear–neutral	1.713	71	1
fear–pride	13.132	71	< 0.001***
fear–sadness	9.073	71	< 0.001***
fear–embarrassment	4.251	71	0.001**
fear–anger	2.993	71	0.079
happiness–neutral	−1.482	71	1
happiness–pride	8.894	71	< 0.001***
happiness–sadness	5.618	71	< 0.001***
happiness–embarrassment	1.624	71	1
happiness–anger	−0.849	71	1
neutral–pride	9.606	71	< 0.001***
neutral–sadness	5.619	71	< 0.001***
neutral–embarrassment	2.551	71	0.272
neutral–anger	0.642	71	1
pride–sadness	−3.666	71	0.010*
pride–embarrassment	−6.188	71	< 0.001***
pride–anger	−10.250	71	< 0.001***
sadness–embarrassment	−2.579	71	1
sadness–anger	−5.461	71	< 0.001***
embarrassment–anger	−2.037	71	1

* *p* < 0.05, **p < 0.01, ***p < 0.001.

**Figure 3.**
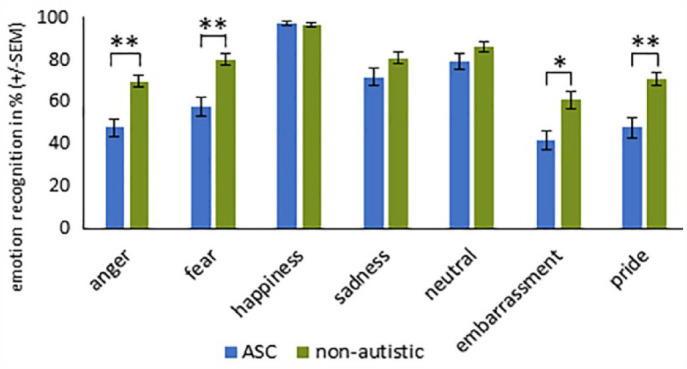
Recognition accuracy (%) for each emotion ± standard error of the mean (SEM) for stimuli with sunglasses in participants with and without ASC. ***Significance at *p* < 0.001. **Significance at *p* < 0.01. *Significance at *p* < 0.05 (with Bonferroni correction).

For the stimuli with sunglasses, the main effect EMOTION (*F*_(6, 65)_ = 79.438, *p* < 0.001, η_*p*_^2^ = 0.880), the interaction EMOTION × GROUP (*F*_(6, 65)_ = 6.246, *p* < 0.001, η_*p*_^2^ = 0.366) as well as the GROUP effect (*F*_(1, 70)_ = 29.564, *p* < 0.001, η_*p*_^2^ = 0.297) reached significance. The two-tailed *t*-test (Bonferroni-corrected) for the interaction EMOTION × GROUP revealed that individuals with ASC had greater difficulty recognizing the emotions fear, pride, embarrassment, and anger than non-autistic participants (see Table 3 und Figure 3).

**Table 3.** Results of the two-tailed, independent samples *t*-tests (Bonferroni-corrected) performed on the interaction EMOTION × GROUP for stimuli with sunglasses.

	*T*	*df*	*p*
Fear	−4.126	70	0.001**
Happiness	0.475	70	1
Neutral	−1.532	70	0.911
Pride	−3.999	70	0.001**
Sadness	−1.825	70	0.51
Embarrassment	−3.236	70	0.013*
Anger	−4.280	70	0.005**

* *p* < 0.05, **p < 0.01, ***p < 0.001.

**Figure 4.**
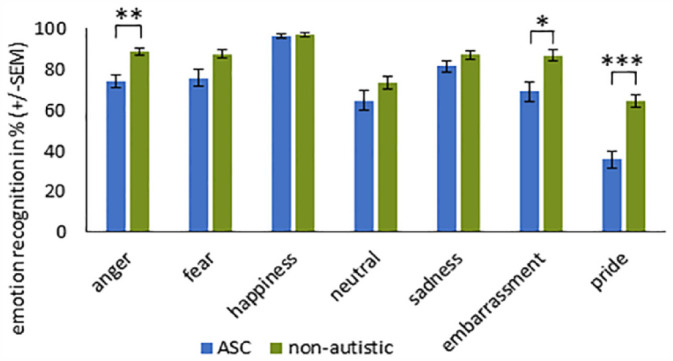
Recognition accuracy (%) for each emotion ± SEM for uncovered stimuli in participants with and without ASC. ***Significance at *p* < 0.001. **Significance at *p* < 0.01. *Significance at *p* < 0.05 (with Bonferroni correction).

For the uncovered stimuli, the main effect EMOTION (*F*_(6, 65)_ = 50.350, *p* < 0.001, η_*p*_^2^ = 0.823), the interaction EMOTION × GROUP (*F*_(6, 65)_ = 4.272, *p* < 0.001, η_*p*_^2^ = 0.283) as well as the GROUP effect (*F*_(1, 70)_ = 32.053, *p* < 0.001, η_*p*_^2^ = 0.314) reached significance. Individuals with ASC showed reduced recognition accuracy of the emotions pride, embarrassment, and anger compared to non-autistic participants (see Table 4 and Figure 4).

**Table 4.** Results of the two-tailed, independent samples *t*-tests (Bonferroni-corrected) performed on the interaction EMOTION × GROUP for uncovered stimuli.

	*T*	*df*	*p*
Fear	−2.690	70	0.068
Happiness	−0.433	70	1
Neutral	−1.601	70	0.802
Pride	−5.391	70	< 0.001***
Sadness	−1.607	70	0.787
Embarrassment	−3.224	70	0.015*
Anger	−3.858	70	0.002**

* *p* < 0.05, **p < 0.01, ***p < 0.001.

**Figure 5.**
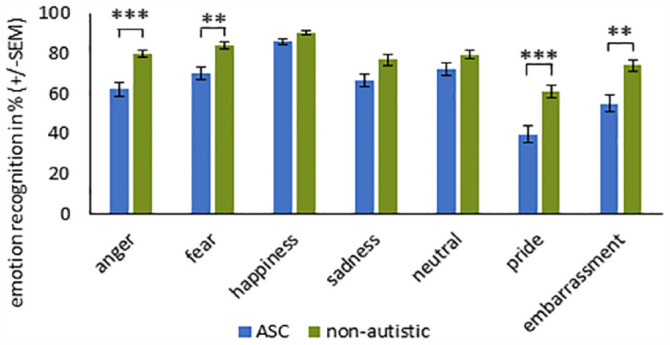
Recognition accuracy (%) across the two groups (± SEM) with the results of the *t*-tests to the significant interaction EMOTION × GROUP (collapsing across condition). ***Significance at *p* < 0.001. **Significance at *p* < 0.01. *Significance at *p* < 0.05 (with Bonferroni correction).

We also performed post hoc *t*-tests (with Bonferroni correction) for the significant interaction EMOTION × GROUP (see Figure 5). Individuals with ASC had greater difficulties categorizing the emotions anger (*t*_(70)_ = –4.983, *p* < 0.001), fear (*t*_(70)_ = –3.630, *p* = 0.005), pride (*t*_(70)_ = –4.466, p < 0.001), and embarrassment (*t*_(70)_ = –3.789, *p* = 0.002).

### Influence of alexithymia and AQ scores

To assess the effect of alexithymia and AQ score, an analysis of covariance was carried out with alexithymia and AQ score as covariates. Emotion recognition was the dependent variable and CONDITION (three levels: uncovered, face mask, and sunglasses), EMOTION (seven different emotions), as well as GROUP (two levels) were the independent variables. The results revealed no significant effect of alexithymia (*F*_(1, 68)_ = 3.207, *p* = 0.078, η_*p*_^2^ = 0.045) or AQ score (*F*_(1, 68)_ = 0.034, *p* = 0.854, η_*p*_^2^ = 0.001). Even though neither covariate was statistically significant, alexithymia did at least exhibit a trend (*p* = 0.078). The interactions, including alexithymia or AQ score failed to reach statistical significance (CONDITION × AQ score: *F*_(1, 68)_ = 0.717, *p* = 0.400, η_*p*_^2^ = 0.012; CONDITION × alexithymia: *F*_(1, 68)_ = 0.408, *p* = 0.525, η_*p*_^2^ = 0.006; EMOTION × AQ score: *F*_(1, 68)_ = 0.664, *p* = 0.418, η_*p*_^2^ = 0.024; EMOTION × alexithymia: *F*_(1, 68)_ = 0.737, *p* = 0.394, η_*p*_^2^ = 0.094; CONDITION × EMOTION × AQ score: *F*_(1, 68)_ = 0.033, *p* = 0.855, η_*p*_^2^ = 0.124; CONDITION × EMOTION × alexithymia: *F*_(1, 68)_ = 0.544, *p* = 0.463, η_*p*_^2^ = 0.183).

### Confusion matrices

**Figure 6.**
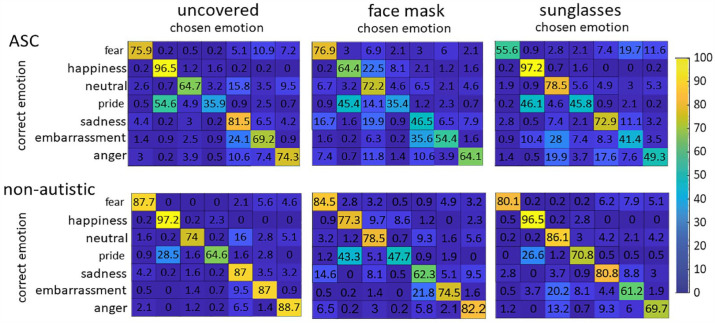
Confusion matrices of correct and perceived/chosen emotions. Percentages compile up to 100% for each correct emotion.

Six confusion matrices, one for each group and condition, were created to show which emotion had been misidentified for another (see Figure 6). We performed the analysis of confusion patterns in a hypothesis-driven manner to avoid computing too many tests. To compare the percentage of correctly detected emotion with the corresponding incorrectly recognized emotion, we performed two-tailed *t*-tests (Bonferroni-corrected). We assumed that “upper-face” expressions are mainly mutually misinterpreted for stimuli with sunglasses. Since in our study, happiness is the only “lower-face” expression, we assumed that confusion in the face-masked stimuli would be highest with the second positive emotion, pride. The results (see Table 5) revealed the same pattern of confusion in both groups.

**Table 5.** Results of the two-tailed *t*-tests (Bonferroni-corrected) performed on the confusion matrices. Indicated are the values for *t*-test between percentages of correctly answered emotion vs incorrectly chosen emotion.

Condition	Correctly chosen emotion	Incorrect chosen emotion	ASC			Non-autistic		
			*T*	*df*	*p*	T	*df*	*p*
Mask	Happiness	Pride	13.215	35	< 0.001***	17.416	35	< 0.001***
Mask	Pride	Happiness	−1.385	35	1	0.652	35	1
Sunglasses	Fear	Sadness	8.634	35	< 0.001***	19.421	35	< 0.001***
Sunglasses	Fear	Anger	7.210	35	< 0.001***	20.369	35	< 0.001***
Sunglasses	Sadness	Fear	15.351	35	< 0.001***	22.593	35	< 0.001***
Sunglasses	Sadness	Anger	14.546	35	< 0.001***	24.694	35	< 0.001***
Sunglasses	Anger	Fear	10.746	35	< 0.001***	22.370	35	< 0.001***
Sunglasses	Anger	Sadness	4.597	35	< 0.001***	15.876	35	< 0.001***

ASC: autism spectrum condition.

* *p* < 0.05, **p < 0.01, ***p < 0.001.

### Reaction time

For the log-transformed reaction times, we conducted a 2 × 3 × 7 repeated-measures mixed-factorial ANOVA with GROUP as a between-group variable, and CONDITION and EMOTION as within-group variables (Table 6). The main effects CONDITION (*F*_(2, 55)_ = 15,523; *p* < 0.001, η_*p*_^2^ = 0.034) and EMOTION (*F*_(6, 51)_ 45,542; *p* < 0.001, η_*p*_^2^ = 0.741) as well as the interaction CONDITION × EMOTION (*F*_(12, 54)_ = 10,562; *p* < 0.001, η_*p*_^2^ = 0.546) reached significance, but no interaction including the between-group variable.

**Table 6.** Results for the eye movement data during the emotion recognition task.

Time window	Condition	ASC	Non-autistic
1	Uncovered	0.07528 ± 0.0370	0.06888 ± 0.0399
	Mask	0.08140 ± 0.0335	0.05401 ± 0.0370
	Sunglasses	0.08198 ± 0.0391	0.07111 ± 0.0368
2	Uncovered	0.08918 ± 0.0575	0.02513 ± 0.0525
	Mask	0.14012 ± 0.0627	0.07656 ± 0.0580
	Sunglasses	−0.00101 ± 0.0613	0.00128 ± 0.0528
3	Uncovered	−0.10379 ± 0.0822	−0.11631 ± 0.0661
	Mask	0.23270 ± 0.0800	0.21410 ± 0.0653
	Sunglasses	−0.30545 ± 0.0702	−0.23093 ± 0.0592

Shown are the means ± SEM of all emotions of a condition for the three time windows used for statistical analysis (32 participants in the ASC group and 31 in the non-autistic group). ASC: autism spectrum condition; SEM: standard error of the mean.

### Fixation patterns

**Figure 7.**
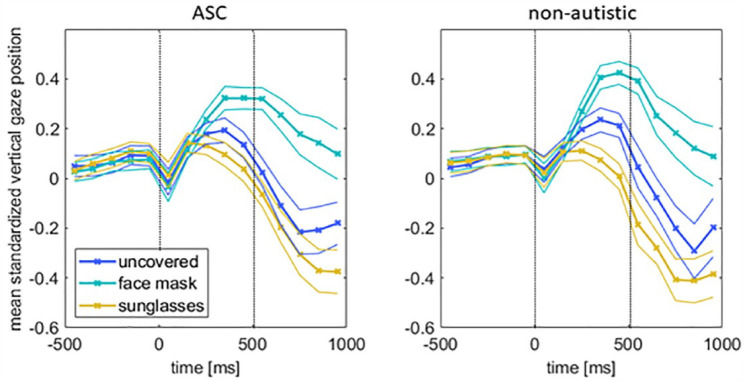
Eye movement data during the emotion recognition task. Shown are the averaged data (± SEM) of all emotions of a condition for the three time windows.

To analyze different fixation patterns, we averaged the vertical gaze positions from our eye tracking data for all emotions in the three time windows (Figure 7). We conducted a 2 × 3 × 3 repeated-measures ANOVA (GROUP, CONDITION, and TIME WINDOW). The main effect of TIME was not significant (*F*(60, 2) = 2.073, *p* = 0.135, η_*p*_^2^ = 0.065), but the main effect of CONDITION (*F*_(2, 60)_ = 30.162, *p* < 0.001, η_*p*_^2^ = 0.5011) as well as the interaction CONDITION × TIME (*F*_(4, 58)_ = 30.275, *p* < 0.001, η_*p*_^2^ = 0.676) were significant. The interactions TIME × GROUP (*F*_(2, 60)_ = 0.145, *p* = 0.865, η_*p*_^2^ = 0.005), CONDITION × GROUP (*F*_(2, 60)_ = 1.291, *p* = 0.283, η_*p*_^2^ = 0.041), as well as the triple interaction CONDITION × TIME × GROUP (*F*_(4, 58)_) = 0.948, *p* = 0.443, η_*p*_^2^ = 0.061) failed to reach statistical significance.

### Emotion recognition questionnaire

An exploratory factor analysis was conducted to assess participants’ subjective ratings of emotion recognition in the pandemic. Data analysis was conducted in R, using psych package (Revelle, 2023). The Kaiser criterion, scree-plot, eigenvalues, and clinical evaluation of the factor structures all suggested three factors as the most parsimonious solution (see Supplementary Material for factor loading plot). We named the three factors: (1) difficulties in emotion recognition, (2) focusing on parts of the body, and (3) maintaining spatial distance. We subsequently conducted two-sample *t*-tests for all three factors. For factor 1, individuals with ASC reported significantly higher scores (*t*(62) = 12.441, *p* < 0.001) as an indication that individuals with ASC self-reported greater difficulty in emotion recognition. There was no group difference for factor 2 (*t*(70) = –1.325, *p* = 0.569) and factor 3 (*t*(67) = –1.126, *p* = 0.793).

## Discussion

### Emotion recognition in individuals with ASC

Despite considerable interest in the extent to which individuals with ASC have difficulties in FER, conflicting results on this issue remain. Our results revealed that FER is reduced in individuals with ASC in comparison to non-autistic individuals, consistent with previous research (Black et al., 2020; Loth et al., 2018). For all three stimulus types (uncovered, face mask, and sunglasses), individuals with ASC had greater difficulty identifying emotions. However, contrary to our assumption, we found no evidence that individuals with ASC found it more difficult to recognize emotions when they had to look at the eyes because the face is wearing a mask, than with uncovered faces.

Individuals with ASC scored lower than the control group in the recognition of masked stimuli. We used emotions that are mainly recognized from the eyes or the mouth and complex emotions, but the interaction EMOTION × GROUP failed to reach statistical significance for face masked stimuli, indicating that “upper-face” and “lower-face” expressions did not influence the different FER between the two study groups for masked stimuli.

When faces were uncovered, individuals with ASC had greater difficulty recognizing pride, embarrassment, and anger than non-autistic participants. The result for anger is in line with previous studies (Ashwin et al., 2006; Philip et al., 2010). Embarrassment is a feeling of discomfort, which usually arises when social norms are violated (Tangney et al., 1996). Individuals with ASC are often not aware of this mostly implicit set of rules and must first acquire it explicitly by observing other individuals, explaining the general accuracy decline (Heerey et al., 2003). The same result was seen for pride, while this result deviated from previous literature, where children with ASC only had to indicate whether they had recognized pride or not (Tracy et al., 2011).

Our results for stimuli with sunglasses revealed a reduced recognition of fear, anger, pride, and embarrassment in ASC than in non-autistic participants. So far, emotion recognition in individuals with ASC when only information from the lower half of the face was available has only been studied in general, not for individual emotions (Baron-Cohen et al., 1997; Sawyer et al., 2012). For the emotions fear and anger, the upper half of the face is considered to convey important information (Wegrzyn et al., 2017). Our findings show that information from only the lower half of the face is insufficient for individuals with ASC to distinguish both emotions and non-autistic people. For fear, this was only the case when the upper half of the face was partially covered, while anger was recognized differently by both groups for the stimuli with sunglasses and uncovered ones.

Collapsing across the three conditions, individuals with ASC revealed a reduced accuracy of the emotions anger, fear, pride, and embarrassment compared to non-autistic participants. For fear and anger, this result is in line with previous finding (Philip et al., 2010), whereas evidence for the complex emotions of pride and embarrassment is limited in adults with ASC.

We found no group differences for happiness and sadness in any of our analyses. In terms of happiness, there may be a ceiling effect for both groups since it is more readily recognized than other emotions (called “Happy Face Advantage;” Kirita & Endo, 1995; Shimamura et al., 2006), which is why previous studies also did not find any group differences (Philip et al., 2010; Rump et al., 2009). Our findings on sadness conflict with prior research that indicated lower perception in individuals with ASC compared to non-autistic people (Boraston et al., 2007; Corden et al., 2008).

Gehdu et al. (2023) recently demonstrated that for masked and uncovered faces, only high-alexithymic autistic participants showed significant difficulties in FER, whereas, there was no difference between non-autistic and low-alexithymic autistic participants. The difficulties in emotion recognition could thus be attributed to alexithymia and not to autism *per se*. Although our analysis did not show a significant influence of alexithymia on FER, there was at least a statistical trend. Even though statistical trends should not be overinterpreted, we report that this finding based on previous research on the relevance of alexithymia to emotion recognition in individuals with ASC (Bird & Cook, 2013; Ola & Gullon-Scott, 2020). In contrast to the study of Gehdu et al. (2023), we could not do a subanalysis, as our sample size was smaller, and in our study, only five participants with ASC fulfilled the criteria for high alexithymia.

The widespread wearing of face masks may be reminiscent of a live version of the “reading the mind in the eyes” test (Baron-Cohen, Wheelwright, Hill, et al., 2001), where individuals with ASC are known to have difficulties recognizing emotions via the eyes. Two studies compared the recognition of basal and complex emotions in individuals with and without ASC when either full faces or only eyes or mouths were presented (Baron-Cohen et al., 1997; Sawyer et al., 2012). Results were conflicting as to whether individuals with ASC were less accurate in FER when viewing only eyes in comparison to the full face. One explanation for the contradictory results could be different paradigms, as in the study by Baron-Cohen et al. (1997), revealing a group difference, there was a two-word response format, which could simplify the recognition of emotions based on whole faces and thus lead to a ceiling effect for this condition, in contrast to the eyes-only condition (Sawyer et al., 2012). In our study, participants had to choose between seven alternative answers, and the condition had no effect on group differences in emotion perception confirming the results of Sawyer et al. (2012). The two studies cited differ from ours in that they presented the mouth or eyes only, whereas, we always showed the entire face, but covered certain parts. This gave participants a choice of which facial area to look at to identify an emotion (see next section).

### Eye tracking

Previous research has mainly revealed that people with ASC have a decreased focus on the eyes when perceiving emotions compared to other facial areas (Black et al., 2020; Corden et al., 2008; Kliemann et al., 2010). Surprisingly, however, a systematic review indicated that this gaze pattern was not consistent across studies, but rather appeared in adulthood since the stimuli may not have been interesting enough for children, or that different developmental steps may not manifest themselves until adolescence (Black et al., 2017).

In our study, we tested adults with and without ASC in FER and used eye tracking to analyze new visual routines in FER when faces are partially covered. We showed that individuals with ASC found it more difficult to recognize emotions, but there was no evidence for different gaze behaviors in comparison to individuals without ASC, so that, we could not confirm the eye-avoidance hypothesis (Tanaka & Sung, 2016). This suggests that lower accuracy of individuals with ASC in emotion recognition, at least for our paradigm, cannot be explained by less fixation on the eyes. Previous studies (see Black et al., 2017 for a review) demonstrating reduced focus on the eye region in individuals with ASC used whole faces without partial occlusion in contrast to our study, which may explain differences in gaze patterns.

Despite numerous studies demonstrating alternative visual strategies in adults with ASC, other studies could not find any group difference. Rutherford and Towns (2008) found that individuals with and without ASC looked longer at the eyes than at the mouth when presenting simple and complex emotions, but there was no difference to control group in overall looking time at eyes. However, there was also no group difference in FER, so that, it is not possible to determine whether different performances in emotion recognition can be attributed to different gaze patterns.

Even before the pandemic, an eye tracking study (Sawyer et al., 2012) analyzed gaze patterns in individuals with and without ASC when basal and complex emotions were presented in three different conditions (full face, eyes only, and mouth only). Individuals with ASC were less accurate at recognizing emotions, but neither study group showed a preference in gaze sequence for one facial region. In line with this study, our findings indicate that even when people with ASC look at identical parts of the face during emotion recognition, they are less accurate. Our results extend those of Sawyer et al. (2012), as the presentation of eyes or mouth only prevents participants from looking at anything else than the isolated face region. So, it remains unclear whether a lack of preference for a facial region in the eye tracking is not due to the stimulus type. We were able to avoid this limitation by presenting the whole face, but reducing the information by partially covering the face, which still allowed participants to look at other regions.

As a restriction, it must be noted that it cannot be ruled out that the absence of distinct gaze patterns across the two research groups is attributable to established adjustment processes. Because mask wearing had been mandatory for several months prior to study entry, and many people with ASC exhibit a variety of strategies that help them camouflage some of their variations in social interaction and mimic non-autistic people’s behaviors to fit in (Alaghband-Rad et al., 2023; Allely, 2019), they may have already become accustomed to look into the eyes for masked stimuli. But, such social camouflage is associated with stress, generalized anxiety, depression, and social anxiety (Cage & Troxell-Whitman, 2019; Hull et al., 2021). Even though we did not investigate it in our study and hence remains speculative, mask-wearing during the pandemic may have been a bigger burden for individuals with ASC, albeit this is not represented in our eye-tracking or behavioral data. However, participants in our study rated their experience of emotion recognition during the pandemic using a questionnaire. The results revealed that individuals with ASC self-reported greater difficulty in recognizing emotions than non-autistic participants.

As a caveat, we used static photographs as stimuli. Studies in non-autistic participants have shown a reduction in accuracy by masks for various basal emotions when only the face was shown (Carbon, 2020; Kastendieck et al., 2022), whereas, this was only the case for happiness when the whole body was presented (Ross & George, 2022). But in general, the use of photographs for studies on FER is not ecological, as in the real world facial emotions are not static entities, but embedded in an interpersonal situation and constantly moving (Foulsham, 2020), while facial, vocal and bodily cues are presented at the same time.

### Underlying mechanisms for the present findings on emotion recognition in individuals with ASC

What can be the responsible mechanisms for the FER decline in ASC? Although our analysis did not show a significant influence of alexithymia on emotion recognition, there was at least a trend. Thus, there is an indication that difficulties in recognizing one’s own emotions were related to FER ability.

Another possible influencing factor of covering face parts on FER is a disruption of holistic face processing (Farah et al., 1998; Young et al., 1987). Faces are typically scanned holistically, taking into consideration the data from every part of the face (Green & Guo, 2018; Wegrzyn et al., 2017), but face mask disrupts holistic processing in non-autistic individuals (Freud et al., 2020). For emotion perception, it was argued that individuals with ASC use an effortful ‘‘systematizing’’ process with explicit cognitive or verbally mediated compensatory mechanisms while non-autistic individuals use more holistic processes (Behrmann et al., 2006; Harms et al., 2010). Our results could not confirm that holistic processing is responsible for our findings, as individuals with ASC showed lower emotion recognition accuracy for all three conditions. Second, eye-tracking data revealed no group difference in fixation patterns. Therefore, a divergent focus on face parts cannot account for the varied emotion recognition. Our groups did not differ significantly in reaction times, age, or IQ, so that, these variables cannot account for the disparities in emotion categorization. In addition to the influence of alexithymia on differential emotion recognition, individuals with ASC may adopt a distinct approach to face processing. Future studies that are specifically designed to evaluate these differences would be interesting.

## Limitations

The largest limitation concerns the sample size, especially for an analysis of the eye-tracking data. This number was partly due to the pandemic, as everyone was asked to separate during that time and eye tracking required the personal presence of participants, making recruitment difficult. At the same time, it was important to us that participants had a professional diagnosis of ASC, which further complicated recruitment.

## Conclusion

This study provides evidence for reduced emotion recognition from the full face, eye region and mouth region in individuals with ASC in comparison to non-autistic individuals. Because individuals with ASC often looked less at the eye region, we hypothesized poorer emotion recognition in masked faces compared to uncovered faces, but this was not confirmed by our results. There were group differences in emotion recognition across all three conditions (uncovered, face mask, and sunglasses), but we found no evidence that individuals with ASC avoid the eye region. So, the disadvantages of individuals with ASC in emotion recognition are not reflected in less fixation on the eyes. Our results are not only relevant with respect to emotion recognition of individuals with ASC during the COVID-19 pandemic, but also add to previously established research on the relationship between emotion recognition and fixation patterns. Regarding alexithymia, we only found a trend as an indication that difficulties in perceiving one’s own emotions might have an influence on emotion recognition. Our data suggest different processing of faces between individuals with and without ASC, which is not as effective in emotion recognition.

## Supplemental Material

sj-docx-2-aut-10.1177_13623613231203306 – Supplemental material for Emotion recognition in autism spectrum condition during the COVID-19 pandemic Supplemental material, sj-docx-2-aut-10.1177_13623613231203306 for Emotion recognition in autism spectrum condition during the COVID-19 pandemic by Tim Schnitzler, Christoph Korn, Sabine C. Herpertz and Thomas Fuchs in Autism

sj-png-1-aut-10.1177_13623613231203306 – Supplemental material for Emotion recognition in autism spectrum condition during the COVID-19 pandemic Supplemental material, sj-png-1-aut-10.1177_13623613231203306 for Emotion recognition in autism spectrum condition during the COVID-19 pandemic by Tim Schnitzler, Christoph Korn, Sabine C. Herpertz and Thomas Fuchs in Autism
