# Supplementary material for: Emotion recognition in autism spectrum condition during the COVID-19 pandemic
Source: Autism. 2023 Oct 26;28(7):1690–702. doi: 10.1177/13623613231203306 (PMC11191665; doi:10.1177/13623613231203306)
Supplement: sj-docx-2-aut-10.1177_13623613231203306 – Supplemental material for Emotion recognition in autism spectrum condition during the COVID-19 pandemic [file sj-docx-2-aut-10.1177_13623613231203306.docx]

**Emotion Recognition Questionnaire**

Below, you will be asked a series of questions, each addressing what has changed for your encounters with other persons since wearing face masks during the Covid 19 pandemic. For most questions, you must place a cross in the appropriate box in each row. For questions 6.2, 6.4, 7.2 and 7.4, please write down the corresponding body parts if you have marked “Neither Agree, nor Disagree (3)”, “Agree (4)” or “Strongly Agree (5)”.

|  | None | 1-2 Persons | 3-4 Persons | 5-8 Persons | > 9 Persons |
| --- | --- | --- | --- | --- | --- |
| - 1. Before the pandemic, with how many people did you meet once every two weeks? |  |  |  |  |  |
| - 1. Since the pandemic, with   how many people have you met once every two weeks? |  |  |  |  |  |

|  | Strongly Disagree (1) | Disagree (2) | Neither Agree, nor Disagree (3) | Agree (4) | Strongly Agree (5) |
| --- | --- | --- | --- | --- | --- |
| 1. I have limited my personal contacts since the beginning of the pandemic. |  |  |  |  |  |
| - 1. Before the pandemic, it was difficult for me to recognize other people’s emotions: |  |  |  |  |  |
| 3.2. Since the beginning of the pandemic with the wearing of face masks it is difficult for me to recognize other people’s emotions: |  |  |  |  |  |
| - 1. Before the pandemic, I maintained physical distance in an encounter with another person: |  |  |  |  |  |
| 4.2. Since the pandemic, I maintain a physical distance in an encounter with another person: |  |  |  |  |  |
| - 1. Before the pandemic, I experienced an emotional distance in personal contact with other people, or I experienced them as less close: |  |  |  |  |  |
| 5.2. Since the pandemic, I experience an emotional distance in personal contact with other people, or I experience them as less close: |  |  |  |  |  |
| - 1. Before the pandemic, I paid more attention to certain parts of the body when meeting other people: |  |  |  |  |  |
| - 1. If you agree, to which ones: |  | | | | |
| - 1. Since the pandemic and wearing of face masks, I pay more attention to certain parts of the body when meeting other people. |  |  |  |  |  |
| - 1. If you agree, to which ones: |  | | | | |
| 7.1. Before the pandemic, I paid more attention to certain parts of the face when meeting other people: |  |  |  |  |  |
| 7.2. If you agree, to which ones: |  | | | | |
| 7.3. Since the pandemic and wearing of face masks, I pay more attention to certain parts of the face when meeting other people. |  |  |  |  |  |
| 7.4. If you agree, to which ones: |  | | | | |
| 8.1. Before the pandemic, I looked other people in the eye: |  |  |  |  |  |
| 8.2. and have experienced them as aversive: |  |  |  |  |  |
| 8.3. Since the pandemic and wearing of face masks, I look other people in the eye: |  |  |  |  |  |
| 8.4. and experience them as aversive: |  |  |  |  |  |
