# Supplementary figures and images for: Emotion recognition in autism spectrum condition during the COVID-19 pandemic
Source: Autism. 2023 Oct 26;28(7):1690–702. doi: 10.1177/13623613231203306 (PMC11191665; doi:10.1177/13623613231203306)

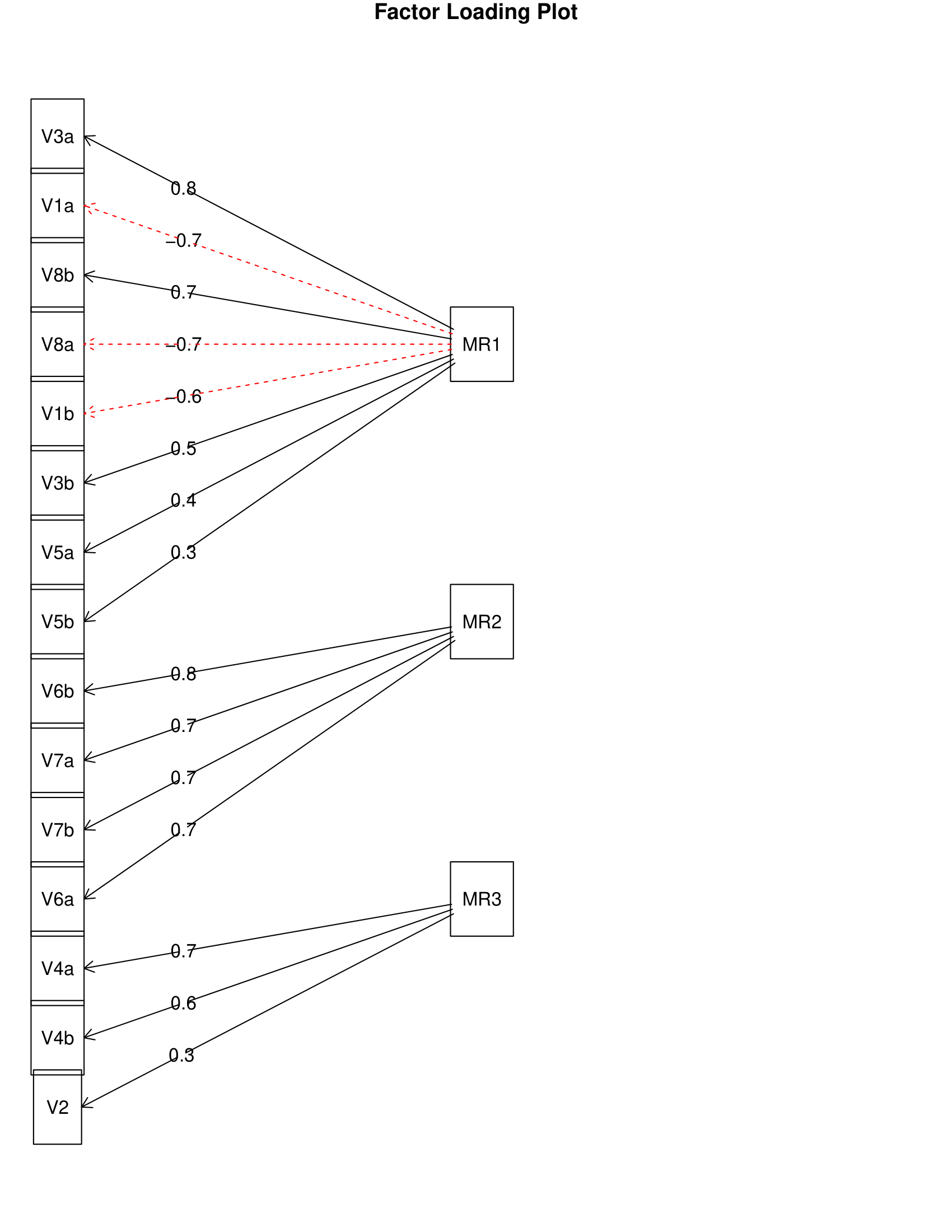

Supplement: sj-png-1-aut-10.1177_13623613231203306 – Supplemental material for Emotion recognition in autism spectrum condition during the COVID-19 pandemic [file sj-png-1-aut-10.1177_13623613231203306.png]
